# Comparative Enhancer Map of Cattle Muscle Genome Annotated by ATAC-Seq

**DOI:** 10.3389/fvets.2021.782409

**Published:** 2021-12-15

**Authors:** Xiukai Cao, Jie Cheng, Yongzhen Huang, Xianyong Lan, Chuzhao Lei, Hong Chen

**Affiliations:** ^1^Key Laboratory of Animal Genetics, Breeding and Reproduction of Shaanxi Province, College of Animal Science and Technology, Northwest A&F University, Yangling, China; ^2^Joint International Research Laboratory of Agriculture and Agri-Product Safety of Ministry of Education of China, Yangzhou University, Yangzhou, China; ^3^College of Animal Science, Xinjiang Agricultural University, Urumqi, China

**Keywords:** cattle, muscle, enhancer, ATAC-seq, GWAS

## Abstract

Annotating regulatory elements could benefit the interpretation of the molecular mechanism of genome-wide association study (GWAS) hits. In this work, we performed transposase-accessible chromatin with sequencing (ATAC-seq) to annotate the cattle muscle genome's functional elements. A total of 10,023 and 11,360 peaks were revealed in muscle genomes of adult and embryo cattle, respectively. The two peak sets produced 8,850 differentially accessible regions (DARs), including 2,515 promoters and 4,319 putative enhancers. These functional elements were associated with the cell cycle, muscle development, and lipid metabolism. A total of 15 putative enhancers were selected for a dual-luciferase reporter assay, and 12 of them showed enhancer activity in cattle myoblasts. Interestingly, the GeneHancer database has annotated the interactions of eight active enhancers with gene promoters, such as embryo-specific peak1053 (log2FC = 1.81, embryo/adult, E/A) with ligand-dependent nuclear receptor corepressor-like protein (*LCORL*) and embryo-specific peak4218 (log2FC = 1.81) with FERM domain-containing 8 (*FRMD8*). A total of 295 GWAS loci from the animal QTL database were mapped to 183 putative enhancers, including rs109554838 (associated with cattle body weight and average daily gain) to peak1053 and rs110294629 (associated with beef shear force and tenderness score) to peak4218. Notably, peak4218 has been found to be involved in mouse embryo development. Deleting peak4218 clearly reduced luciferase activity (*P* = 3.30E-04). Our comparative enhancer map is expected to benefit the area of beef cattle breeding.

## Introduction

Eukaryotic genomes are tightly packaged into chromatin leaving biologically active regions to be accessible to the transcription machinery (1). These open or accessible regions have been found to be the primary positions of functional elements (2). The dynamics of these regions are involved in regulating gene expression and embryo development (3). DNA mutations within the accessible regions, typically referred to as regulatory variations, could affect the functions of promoters and enhancers (4, 5). Therefore, annotating the accessible regions may help to detect functional elements and also bridge the so-called genotype-to-phenotype gap.

Today, the Functional Annotation of Animal Genomes (FAANG) consortium is working to create reference functional maps of farmed animals by profiling the landscape of chromatin accessibility. However, studies regarding the *Bos taurus* cattle muscle genome are still limited (6). With respect to comparative genomics, four studies have identified regulatory elements for cattle and sheep genomes based on ENCODE information (7–10) but did not detail the muscle-specific elements or differentially accessible regions (DARs). More recently, a comparative analysis of the chromatin accessibility in muscle, liver, and hypothalamus of Brahman cattle (*Bos indicus*) has been reported. A total of 11,439 muscle-specific accessible regions were called for three muscle replicates and these peaks were enriched mostly with muscle cell development and myocyte enhancer factor 2 (MEF2) was pointed as a master regulator of muscle-specific open chromatin regions (11). This study used *Bos indicus* cattle as subject which is in different subspecies from *Bos taurus* cattle and focused on only one stage of muscle development. Additionally, the landscape of accessible chromatin in *Bos taurus* cattle early embryos has been studied without focusing on muscle (12).

Transposase-accessible chromatin with sequencing (ATAC-seq) is a well-accepted method for functional genome annotation (13). It is a fast and sensitive alternative to DNase I hypersensitive site sequencing (DNase-seq), micrococcal nuclease sequencing (MNase-seq), and formaldehyde-assisted isolation of regulatory elements (FAIRE), and it requires a much smaller amount of starting material to generate high-fidelity data by employing hyperactive Tn5 transposase (14). Qinchuan cattle (*Bos taurus*) is one of the top five local cattle breeds in China. It is well-known for good endurance and adaptability and has been used as a major labor force in agricultural production for thousands of years (15). Today, however, with the beef demand increasing, Qinchuan cattle is widely used as beef breed and its beef production trait has been a primary focus of researchers.

**Figure 1:**
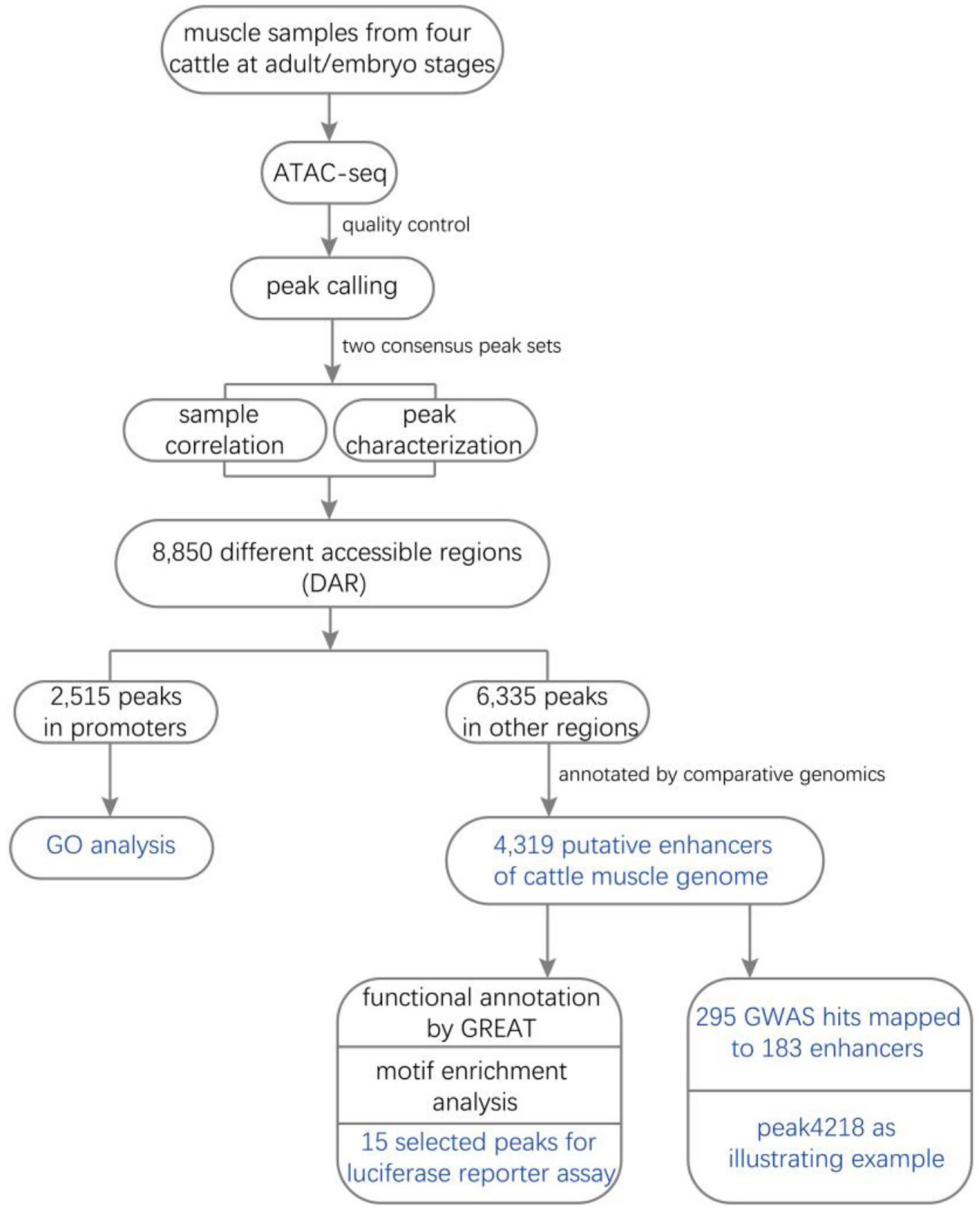
Study roadmap. Four samples of Qinchuan longissimus dorsi from two adult and two embryo cattle were subjected to ATAC-seq. After quality control, we obtained 22-42 million of final read pairs for peak calling. The four peaks produced 10,023 and 11,360 common peaks for adult and embryo groups, respectively. Sample correlation (PCA and correlation coefficients) analyses and peak characterization (peak width, fold enrichment, and genomic distributions) were performed. The two common peak sets were combined to form a union peak set which contained 8,850 DARs and 10,342 stable peaks. We detected 2,515 promoter peaks and 4,319 putative enhancers from the 8,850 DARs. Metascape and GREAT were used to perform GO and functional annotation analyses for promoters and putative enhancers, respectively. Fifteen putative enhancers were selected to be subjected to luciferase reporter assay. A total of 140/3,494 (4.00%) embryo-specific and 43/825 (5.21%) adult-specific putative enhancers were successfully annotated by 295 GWAS hits within ±20 kb, including rs110294629 to peak4218.

Here, to explore the molecular mechanism underlying the muscle growth and development of Qinchuan cattle, we performed ATAC-seq for longissimus dorsi samples from two adult and two embryo cattle. The comparative enhancer map was generated according to the flowchart in Figure 1. From 8,850 DARs, a total of 2,515 promoter peaks and 4,319 putative enhancer peaks were identified, and the latter was integrated with genome-wide association study (GWAS) hits. Additionally, we used a dual-luciferase reporter assay to determine enhancer activity and found that deleting embryo-specific peak4218 (log2FC = 1.81) clearly reduced such activity. These results may provide further insights into the genetic architecture of cattle muscle development.

## Materials and Methods

### Animals

The experimental protocols were approved by the Institutional Animal Care and Use Committee of Northwest A&F University (NWAFAC1023). Longissimus dorsi samples were collected from four female Qinchuan cattle (two adults at about 2.5 years old and two embryos at about 2.5 months old) in slaughterhouses (Shaanxi and Gansu provinces). The two adult cattle (C-6-2, D-1-1) were commercial individuals and genetically unrelated. After mating, the adult cattle were fed on corn-corn silage diet. The two female embryos (T-6-2, T-7-1) were obtained by slaughtering the adult cattle and their ages were estimated based on the mating time. Four samples of longissimus dorsi were collected.

### Library Preparation of ATAC-Seq

The ATAC-seq library was prepared according to an improved protocol for frozen tissue as described previously (14). Briefly, (1) 20 mg frozen longissimus dorsi were thawed and treated to suspend individual cells; (2) cells were isolated with cell lysis buffer and the nuclei (~50,000) were purified with higher-speed centrifugation; (3) purified nuclei were processed for Tn5-mediated tagmentation and adapter incorporation; (4) the tagmented DNA was purified for PCR amplification; (5) a DNA-based fluorometric assay and automated capillary electrophoresis were used to assess the library qualities. The constructed libraries were subjected to next-generation sequencing on a HiSeq2500 Illumina sequencer with PE 150 mode. The raw sequencing reads have been submitted to NCBI (PRJNA555664).

### Data Quality Control and Peak Calling

Adapters and low-quality (phred quality < 10) bases were removed from raw sequencing reads with Trimmomatic (version 0.38) (16), and trimmed reads were then aligned to cattle reference genome (ARS-UCD 1.2) with bowtie2 (version 2.4.3) (17). High-quality paired alignments (mapping quality ≥ 30) were extracted with samtools v1.9 after filtering unmapped reads, mate unmapped reads, non-primary alignments, and reads failing platform. To generate valid pairs for peak calling, PCR duplications and mitochondria reads were further removed by Picard (version 1.126) and bedtools (version 2.26.0) with default parameters, respectively (18). The distribution of insertion size was plotted with R program (version 3.5.1) to evaluate chromatin integrity. MACS2 was used for peak calling with the following parameters: “–nomodel –shift−75 –extsize 150” (19). The irreproducible discovery rate (IDR) method was used to assess the consistency of individual replicate/pseudoreplicate peak sets (20). The peaks of two replicates were merged using idr (version 2.0.2) (https://github.com/nboley/idr). Next, the two common peak sets were combined to form a union peak set according to the criteria that individual peaks were merged if overlapping within 300 bp using bedtools (version 2.26.0) with parameter of “bedtools multiinter” followed by “bedtools merge –d 300.” The number of reads of each sample at the union intervals were re-called with the parameter of “bedtools multicov -bams” to generate count matrix. For each union peak, its enrichment value is defined as the ATAC-seq signal intensity (normalized read count per base) subtracted from the background noise (normalized read count per base). The resulting count matrix was used as input to DESeq2 (version 1.32.0) to call DARs with *P* < 0.05 (21).

### Peak Annotation and GWAS Hits Integration

Peak annotation was carried out by ChIPseeker (22), rtracklayer (23), and GenomicFeatures (24) with the latest cattle genome assembly, ARS-UCD 1.2. The promoter was annotated as ±3 kb of the transcription start site (TSS) with NCBI genome annotation file. To recognize the differentially putative enhancers, DARs were mapped to cattle genomic regulatory regions identified by Nguyen et al. (8) and ENCODE enhancer-like regions (v3) (http://zlab-annotations.umassmed.edu/enhancers/). GWAS hits about production traits and meat and carcass traits were retrieved from Animal QTLdb (http://www.animalgenome.org/cgi-bin/QTLdb/index) and filtered with *P* >0.05 and unavailable breed information. Genome coordinates were converted to ARS-UCD 1.2 with the NCBI Genome Remapping Service (https://www.ncbi.nlm.nih.gov/genome/tools/remap). GWAS hits were mapped to putative enhancers within ± 20 kb.

### Functional Annotation and Motif Enrichment Analysis

Gene ontology (GO) was performed with Metascape for the promoter peaks (25). Metascape is easy to use and has more collected GO items, despite being aimed at only a limited number of organisms (mainly developed for humans and other model animals). Most of our input genes (>90%) could be discerned by Metascape. Functional annotation and motif enrichment analysis for the putative enhancers were performed by GREAT (26) and AME (http://meme-suite.org/tools/ame), respectively.

### Cell Culture

Cattle primary myoblasts were isolated and cultured from embryo longissimus dorsi as described previously (27). These cells were cultured with growth medium comprising DMEM (Gibco) supplemented with 20% FBS (Thermo Fischer Scientific) and 1% double antibiotics at 37°C in 5% CO_2_. HEK293T (human embryonic kidney cell line), C2C12 (mouse myoblast cell line), 3T3-L1 (mouse immortalized preadipocyte), and MAC-T (bovine mammary alveolar cell line) were maintained in our laboratory. These four cell lines grew in 10% FBS and were used for the dual-luciferase reporter assay when they reached approximately 70% confluence at 37°C in 5% CO_2_. The five kinds of cells were used to identify muscle-specific enhancers.

### Luciferase Reporter Assay

With the standard phenol-chloroform method, we extracted genomic DNA from 1 ml 2% heparin-treated whole blood samples. The genomic DNA was used to amplify potential enhancers with the primer pairs listed in Supplementary Table 1. First, we selected 15 well-known genes with breeding potential, and then one candidate enhancer around or within each gene was randomly selected from the enhancer map. The PCR products were cloned into the pGL3-Promoter vector digested by *BamH* I and *Sal* I downstream of the luciferase gene. TurboFect (Thermo Scientific, USA) was used for co-transfection of the pRL-TK and recombinant pGL3-Promoter vectors. Blank pGL3-Basic plasmid (SV40 Promoter/Enhancer^−/−^), blank pGL3-Promoter plasmid (SV40 Promoter/Enhancer^+/−^), and pGL3-Control plasmid (SV40 Promoter/Enhancer^+/+^) were used as the blank, negative, and positive controls, respectively. Cells were collected to detect the luciferase activity with the Dual-Luciferase Reporter Assay System (Promega, USA) after 24-36 h incubation. The luciferase signal was normalized to the *Renilla* luciferase signal. To delete peak4218, we synthesized a 281-bp fragment that combined chr29: 43660064-43660146 and chr29: 43660708-43660906 without the peak4218 region (chr29:43660147-43660707).

### Statistical Analysis

Data were presented as mean ± SD. One-way ANOVA was used to compare the relative luciferase activity of putative enhancers at a significance level of *P* < 0.05.

## Results

### Data Quality Control and Peak Calling

We got 71-83 million of raw read pairs and the alignment rate ranged from 86-88%. The final read number was 23,933,263 for C-6-2 (adult), 22,333,042 for D-1-1 (adult), 30,401,792 for T-6-2 (embryo), and 42,479,978 for T-7-1 (embryo) (Table 1). Due to the low sequencing (about 7×) depth and high PCR duplication (19-52%), it was not surprising that the number of final read pairs for peak calling were <50 million (ENCODE ATAC-seq standard, https://www.encodeproject.org/atac-seq/).

**Table 1:** Summary mapping statistics and quality control per sample.

**Sample**	**Stage**	**Raw** **reads**	**Clean** **reads**	**Mapped** **reads**	**% Mapped** **reads**	**% PCR duplication**	**% mtDNA**	**Final reads**	**% Final reads**	**Peaks**	**% Genome coverage**	**FRiP score**	**Common** **peaks**
C-6-2	Adult	72,033,458	70,474,823	63,697,320	88.43	52.17	3.21	23,933,263	33.23	19,210	0.22	0.08	10,023
D-1-1	Adult	71,631,156	69,157,477	63,873,523	89.17	52.60	4.51	22,333,042	31.18	20,361	0.32	0.10	
T-6-2	Embryo	79,271,255	77,542,120	71,035,710	89.61	35.57	1.11	30,401,792	38.35	23,618	0.33	0.10	11,360
T-7-1	Embryo	83,237,472	83,237,472	76,210,914	91.56	19.66	0.69	42,479,978	51.03	24,550	0.36	0.13	

*FRiP score means fraction of all mapped reads that fall into the called peak regions*.

**Figure 2:**
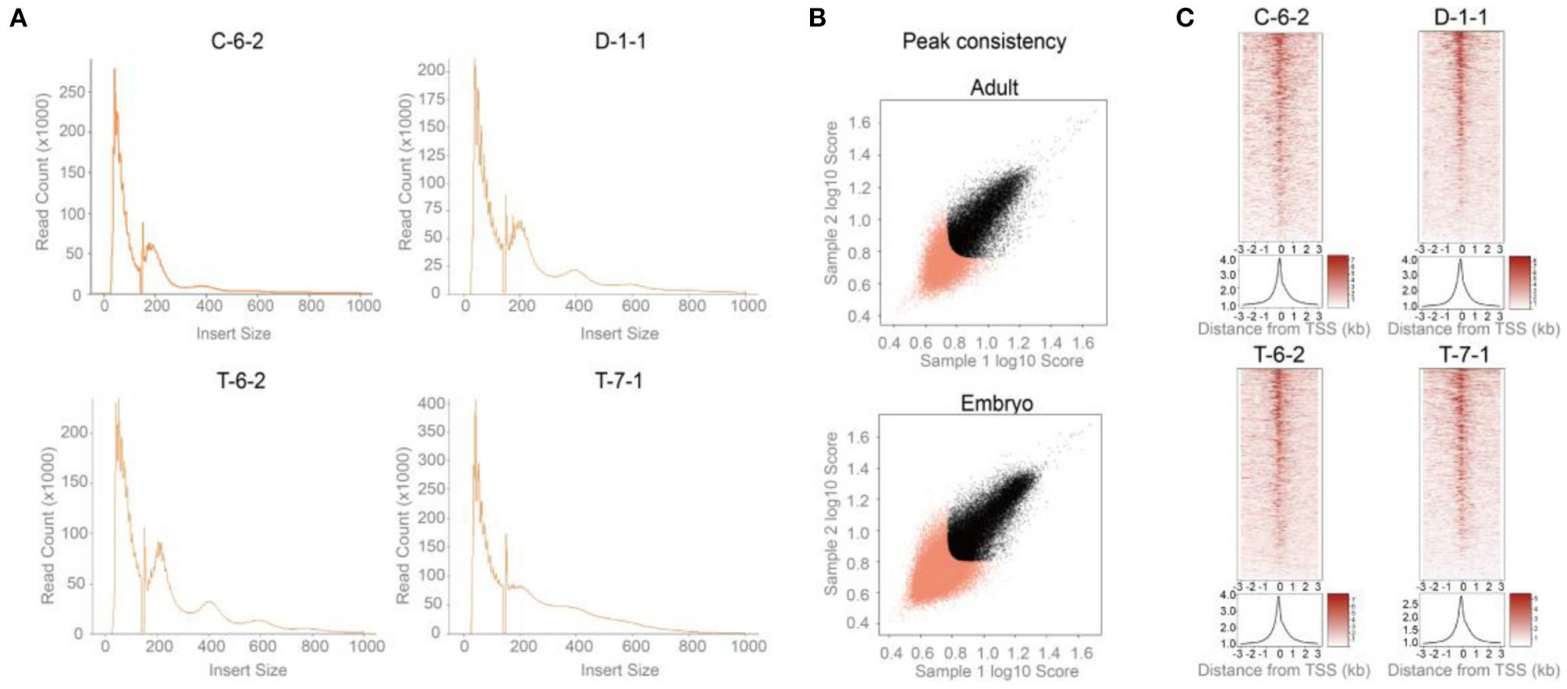
Data quality control and peak detection for ATAC-seq. **(A)** The insert size distribution of sequenced fragments had clear periodicity of ~200 bp. **(B)** Peak consistency between two replicates of each group indicated by IDR. x-Sample 1 log10 peak scores vs. y-Sample 2 log10 peak scores. Sample 1 and 2 are the two replicates of given group. Peaks that did not pass the IDR threshold (0.05) were colored red (https://screen.encodeproject.org/). The self-consistency ratio and rescue ratio of the adult group were 1.20 and 1.82, respectively, and as for the embryo group, they were 1.41 and 2.62, respectively. **(C)** TSS enrichment of ATAC-seq reads. Adult: C-6-2, D-1-1. Embryo: T-6-2, T-7-1.

As shown in Figure 2A, two peaks were observed at <100 and 180-247 bp for all four samples, agreeing with previous reports. The first and second peaks corresponded to nucleosome-free regions and a single nucleosome where Tn5 inserted around, respectively (28). A total of 19,210 (C-6-2), 20,361 (D-1-1), 23,618 (T-6-2), and 24,550 (T-7-1) peaks were detected. The genome coverages of these peak sets ranged from 0.22 to 0.36% (Table 1). After peak calling, IDR was used to evaluate the peak reliability of each sample by producing pseudoreplicate peak sets. The number of reproducible peaks in black roughly accounted for half of the true peak number (Supplementary Figure 1). We further used IDR to measure the peak reproducibility of the two replicates in each group. The self-consistency ratio and rescue ratio of the adult group were 1.20 and 1.82, respectively, and as for the embryo group, they were 1.41 and 2.62, respectively (Figure 2B). The fraction of all mapped reads that fell into identified peaks (FRiP score) was used to roughly evaluate the signal-noise ratio. We found FRiP score range from 0.08 to 0.13 (Table 1). The lower sequencing depth and higher PCR duplication may generate less peaks, which partially leads to higher signal-noise ratio. Additionally, TSS enrichment was also observed for ATAC-seq reads (Figure 2C), which was consistent with previous studies (29). These results suggested that the four ATAC-seq libraries are acceptable but may be not ideal (please refer to the Discussions).

### Peak Characterizations and DAR Detection

**Figure 3:**
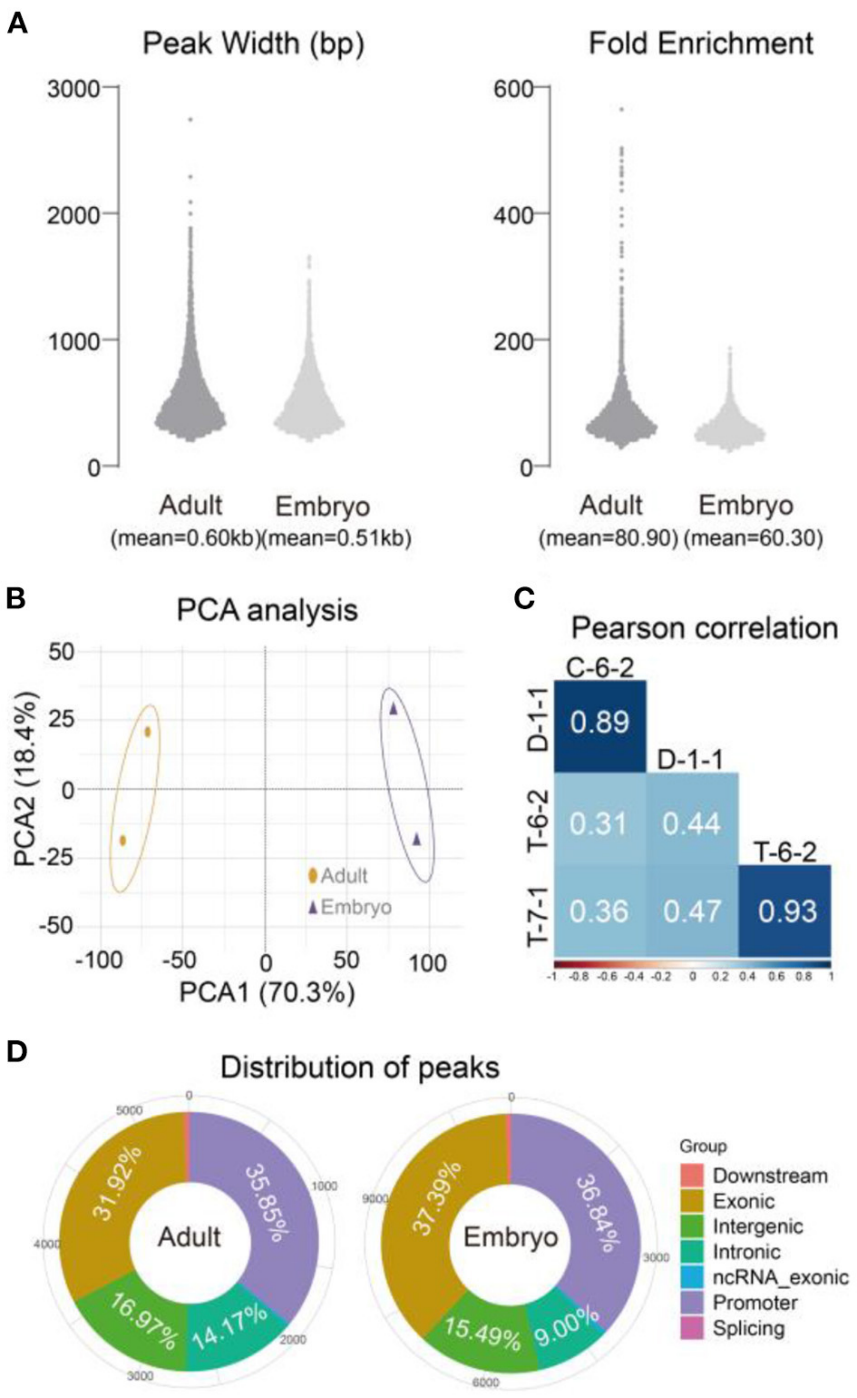
Peak characterizations and sample correlations using two common peak sets. **(A)** Widths and fold enrichments of two common peak sets. **(B)** PCA analysis of four individuals. **(C)** Pearson correlation of four individuals. **(D)** Summary of common peak distributions. Adult: C-6-2, D-1-1. Embryo: T-6-2, T-7-1.

The four peaks produced 10,023 and 11,360 common peaks for adult and embryo groups, respectively (Table 1). The mean peak width was 0.60 kb for adults and 0.51 kb for embryos. Adult peaks appeared to have a higher fold enrichment than embryo peaks (80.90 vs. 60.30) (Figure 3A). An expected correlation was observed with principal component analysis (PCA) and Pearson correlation analyses (Figures 3B,C). Similar genomic distributions were observed for adult and embryo peaks (Figure 3D). Approximately 60% of the accessible regions were unmapped to promoters, suggesting the presence of other functional elements. Peak sets called in each group were combined to form a union peak set which contained 8,850 DARs and 10,342 stable peaks. We used 8,850 DARs (7,422 embryo-specific peaks and 1,428 adult-specific peaks) for the downstream analyses.

### Annotating Promoter Peaks and Putative Enhancer Peaks

We obtained 2,515 promoter peaks from 8,850 DARs (Supplementary Table 2). Some of these peaks were mapped to muscle development candidates, such as early growth response 1 (*EGR1*) (30), kruppel-like factor 15 (*KLF15*) (31), insulin-like growth factor 2 mRNA binding protein 2 (*IGF2BP2*) (32), fatty acid synthase (*FASN*) (33), and fatty acid desaturase 1 (*FADS1*) (34). The 2,515 genes were enriched for the cell cycle (GO:0051301, GO:0010564, R-HAS-1640170, GO:0044839), cellular protein catabolic process (GO:0044257), and muscle development (GO:0061061) (Supplementary Figure 2).

**Figure 4:**
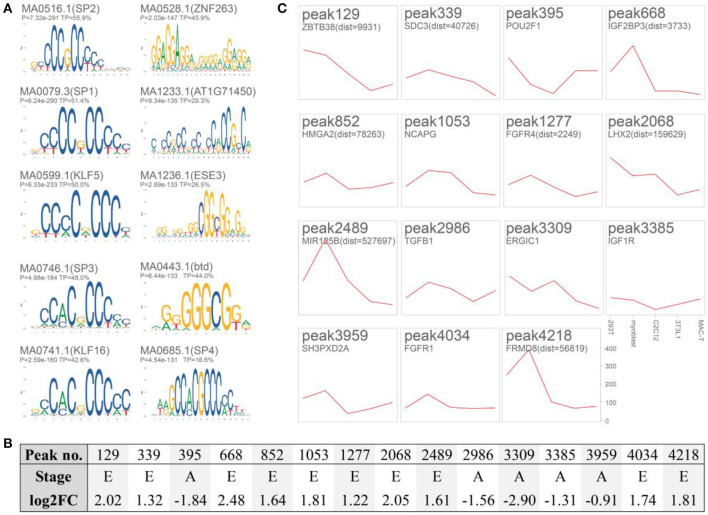
Motif enrichment analysis and validation of 15 putative enhancers. **(A)** Enriched motifs of the 4,319 putative enhancers with AME (Supplementary Table 5). **(B)** Detailed information of the 15 selected putative enhancers. E, embryo-specific; A, adult-specific; log2FC, log_2_ fold change (E/A); **(C)** Twelve putative enhancers cloned into pGL3-Promoter (SV40 Promoter/Enhancer^+/−^) significantly triggered luciferase activity in cattle myoblast (*P* < 0.05, *n* = 5, compared with blank pGL3-Promoter, the raw data and significance were listed in Supplementary Table 6). HEK293T, human embryonic kidney cell line; C2C12 mouse myoblast cell line; 3T3-L1, mouse immortalized preadipocyte; MAC-T, bovine mammary alveolar cell line; myoblast, the isolated myoblasts from Qinchuan embryo muscle.

A total of 4,319 differentially putative enhancers (Supplementary Table 3) were revealed by integrating the remaining 6,335 DARs with functional elements annotated by Nguyen et al. (8) and the ENCODE enhancer-like regions database. These regions were associated with muscle development and lipid metabolism, as revealed by GREAT (Table 2, Supplementary Table 4). Motif enrichment analysis showed that transcription factor families, such as specificity protein (SP) (35) and KLF (36), could bind to these putative enhancers (Figure 4A, Supplementary Table 5). Detailedly, the 3,494 embryo-specific putative enhancers were involved in abnormality of the liver, global developmental delay, and skeletal muscle atrophy (top 20 items of GREAT) (Supplementary Table 4). The 825 adult-specific putative enhancers were annotated in muscle weakness, myopathy, and abnormality of muscle morphology (top 5 items of GREAT) (Supplementary Table 4).

**Table 2:** Top 20 enrichment items of the 4,319 putative enhancers by GREAT functional annotation (detailed information was listed in Supplementary Table 4).

**Term name**	**FDR *Q*-value**	**Observed region hits**	**Region set coverage**
Muscle weakness	1.03E-04	185	4.83E-02
Skeletal muscle atrophy	7.88E-05	119	3.10E-02
Increased serum lactate	3.43E-04	41	1.07E-02
Abnormality of muscle morphology	3.25E-04	280	7.30E-02
Global developmental delay	4.29E-04	347	9.05E-02
Abnormal myelination	4.67E-04	98	2.56E-02
Abnormality of lipid metabolism	5.64E-04	55	1.43E-02
Muscular hypotonia	2.80E-03	344	8.97E-02
Abnormality of skeletal muscles	2.54E-03	145	3.78E-02
Abnormality of muscle size	3.17E-03	148	3.86E-02
Hypertrophic cardiomyopathy	4.65E-03	49	1.28E-02
Narrow vertebral interpedicular distance	4.65E-03	7	1.83E-03
Abnormal CSF metabolite level	4.77E-03	27	7.04E-03
Excessive daytime somnolence	5.01E-03	7	1.83E-03
Abnormality of muscle fibers	4.88E-03	44	1.15E-02
Increased CSF lactate	4.90E-03	25	6.52E-03
Lactic acidosis	4.95E-03	53	1.38E-02
Cardiomyopathy	5.03E-03	99	2.58E-02
Lipid accumulation in hepatocytes	5.09E-03	36	9.39E-03
Abnormality of the liver	5.02E-03	160	4.17E-02

### Integrating GWAS Hits to Putative Enhancers

We selected 15 putative enhancers (log2FC [embryo/adult, E/A] was listed in Figure 4B) by focusing on well-known genes. Only three of them did not trigger the luciferase activity in cattle myoblasts compared to that of the pGL3-Promoter (*P* > 0.05, Figure 4C and raw data and significance were presented in Supplementary Table 6). This result suggested the acceptable reliability of these putative enhancers. As for the remaining peaks, their homologous regions (8/12) in humans could interact with gene promoters as recorded in the GeneHancer database (Supplementary Figure 3) [i.e., peak339 with syndecan 3 (*SDC3*), peak852 with high-mobility group AT-hook 2 (*HMGA2*), peak1053 with ligand-dependent nuclear receptor corepressor-like protein (*LCORL*), peak1227 with fibroblast growth factor receptor 4 (*FGFR4*), peak2068 with NIMA-related kinase 6 (*NEK6*), peak2986 with transforming growth factor β 1 (*TGF*β*1*), peak3959 with SH3 and PX domains 2A (*SH3PXD2A*), and peak4218 with FERM domain containing 8 (*FRMD8*)]. Chromosome conformation capture is still required to confirm these interactions in the cattle muscle genome.

**Figure 5:**
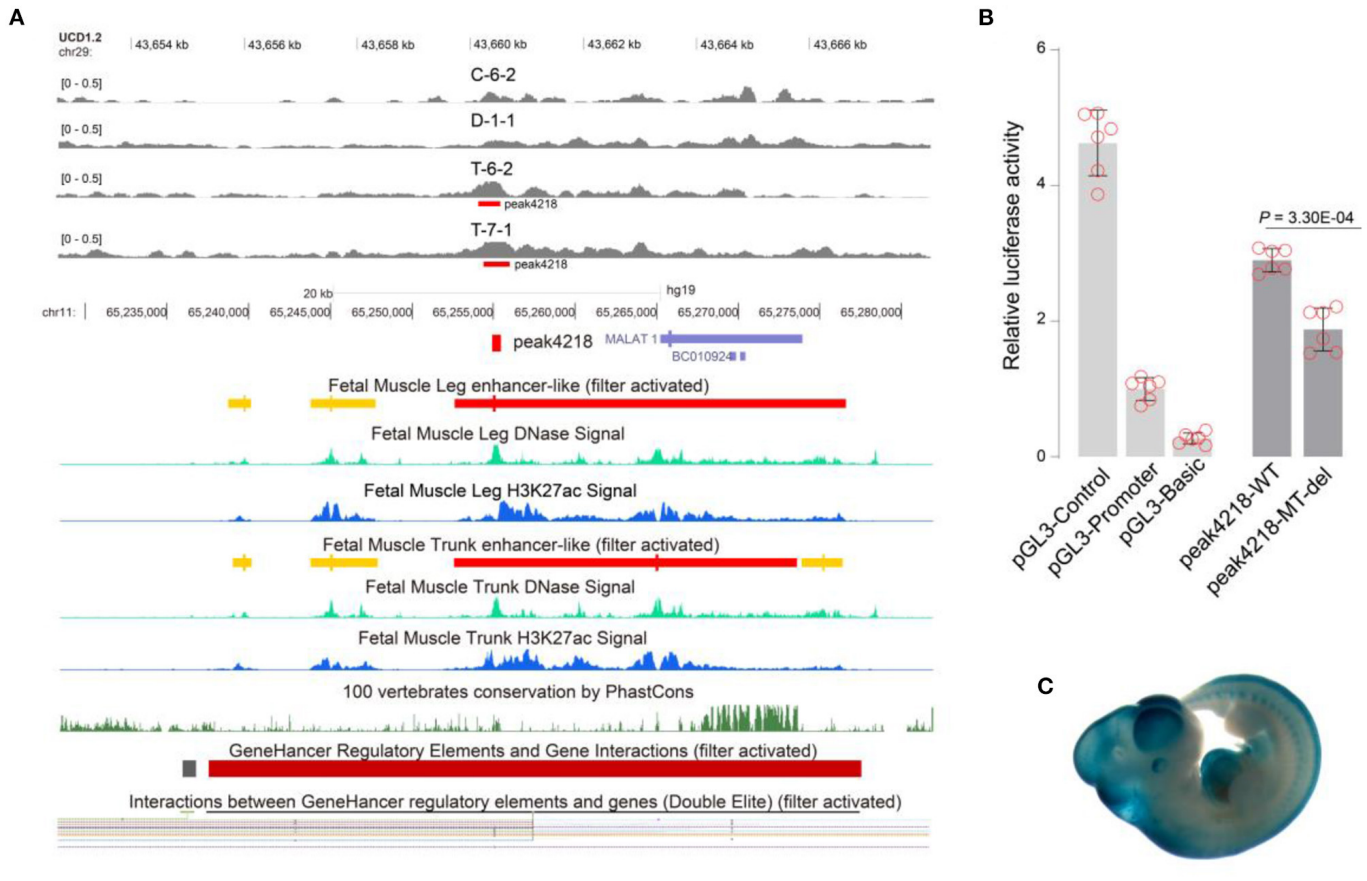
Annotating peak4218 with comparative genomics and the enhancer activities of its mutants. **(A)** Human homologous region of peak4218 acts as enhancers to contact with *FRMD8* promoter predicted GeneHancer database. **(B)** Deleting peak4218 showed clear reduction in luciferase activity (*P* = 3.30E-04). Data were represented as mean ± SD (*n* = 6). **(C)** Homologous peak4218 in mouse genome was involved in embryo development (picture from VISTA Enhancer with ID: hs1759). Peak4218 was embryo-specific with log2FC = 1.81.

A total of 3,635 GWAS hits (Supplementary Table 7) regarding meat and carcass traits and production traits (trait classes of Animal QTLdb) were used to map to the 4,319 enhancers. Finally, 140/3,494 (4.00%) embryo-specific and 43/825 (5.21%) adult-specific putative enhancers were successfully annotated by 295 GWAS hits within ± 20 kb, including rs109554838 to peak1053 and rs110294629 to peak4218 (Supplementary Table 8). The homologous region of peak4218 has been annotated as an enhancer in the human muscle genome (Figure 5A). The embryo-specific peak4218 (log2FC = 1.81) was annotated to be involved in spasticity, hypertonia, liver abnormality (Supplementary Table 4) by GREAT and the GWAS hits mapped to it were associated with beef shear force and tenderness score (Supplementary Table 8). We found that deleting peak4218 clearly reduced luciferase activity (*P* = 3.30E-04) (Figure 5B). Additionally, this region has been found to be implicated in mouse embryo development, as documented in VISTA Enhancer (ID: hs1759) (Figure 5C). These results may help to interpret the GWAS hits underlying cattle muscle development.

## Discussion

Consistent efforts are being made to better understand the genetic architecture of cattle muscle, given the increasing demand for beef. Using comparative genomics, three studies have annotated regulatory elements for cattle genome (*Bos taurus*), but they did not list muscle-specific elements (7–9). More recently, two studies have identified functional elements for beef cattle with ATAC-seq. One of them reported the landscape of accessible chromatin in *Bos taurus* cattle using whole embryo (12). The other study identified a total of 11,439 muscle-specific accessible regions for Brahman cattle (*Bos indicus*) which is in different subspecies from *Bos taurus* cattle (11). To provide a perspective to the regulatory elements of *Bos taurus* cattle muscle genome, four samples of Qinchuan longissimus dorsi from two adult and two embryo cattle were subjected to ATAC-seq.

We obtained 70-80 million of raw read pairs and more than 88% of them could be mapped to the reference genome. This result was consistent with studies on muscle of *Bos indicus* cattle whose mapping rate was ranging from 86 to 88% and studies on pig muscle whose mapping rate was about 89% (11, 37). The percentage of our mapped reads was acceptable as indicated by ENCODE ATAC-seq standard (alignment rate > 80%) (https://www.encodeproject.org/atac-seq/). However, the final reads for peak calling, especially of the adult group, were less than ENCODE ATAC-seq data standard which requires at least 50 million reads for paired-ended sequencing. This result fell into our expectation given the following two reasons: low sequencing depth and high PCR duplication. Our sequencing depth was about 7×, producing about 20 billion raw bases and 70 million raw read pairs. But the ATAC-seq for pig muscle genome were performed at more than 14× sequencing depth which produced more than 40 billion raw bases, 140 million raw read pairs, and 70 million final read pairs (37). It can be inferred that if the sequencing depth reached to 14×, our final reads for peak calling will be doubled. Although the PCR duplication standard is not defined by ENCODE, to our knowledge, it usually ranges from 2 to 30% (11, 37). But the high PCR duplication (51.5%) of ATAC-seq has been reported in chicken lung (37). The number of duplicated reads were closely related with the PCR cycle for library construction (14). In present study, we used a total of 10 PCR cycles (five cycles for pre-amplification of transposed fragments and five cycles for additional cycles) to add on the enough Illumina adapter sequences, which may lead to library over-amplification at this point, especially the “adult” libraries.

Despite of the above findings, our ATAC-seq libraries were qualified to call peaks in consideration of the acceptable values of insert size, IDR, and FRiP according to ENCODE standard or previous reports. The insert size distribution of sequenced fragments from human chromatin had clear periodicity of approximately 200 bp, suggesting many fragments are protected by integer multiples of nucleosomes (28). The distributions of insert size of our libraries showed clear periodicity equal to the helical pitch of DNA. It has been reported that reads below 100 bp were considered nucleosome free, reads between 180 and 247 bp were considered to be mononucleosomes, reads between 315 and 473 bp were considered to be dinucleosomes (28).

The IDR method was developed by Qunhua Li and Peter Bickel's group and is extensively used by the ENCODE and modENCODE projects to evaluate ChIP-seq and ATAC-seq (20). It operates on the replicated peak set and compares consistency of ranks of these peaks in individual replicate/pseudoreplicate peak sets. IDR uses self-consistency ratio and rescue ratio to measure the peak reproducibility. Self-consistency ratio is calculated as max (N1, N2) / min (N1, N2), and rescue ratio is calculated as max (Np, Nt) / min (Np, Nt), where N1 represents replicate 1 self-consistent overlapping peaks (comparing two pseudoreplicates generated by subsampling Rep1 reads), N2 represents replicate 2 self-consistent overlapping peaks (comparing two pseudoreplicates generated by subsampling Rep2 reads), Nt represents true replicate consistent overlapping peaks (comparing true replicates Rep1 vs. Rep2), and Np represents pooled-pseudoreplicate consistent overlapping peaks (comparing two pseudoreplicates generated by subsampling pooled reads from Rep1 and Rep2) (38). In our study, the self-consistency ratio and rescue ratio of the adult group were 1.20 and 1.82, respectively, and as for the embryo group, they were 1.41 and 2.62, respectively. According to ENCODE standard, the experiment will be ideal if both rescue and self-consistency ratios were <2, but it is acceptable that if one value is <2 and the other is more than 2. Previous study about ZBTB33 ChIP-seq used K562 (human erythroleukemic cell line) replicates with self-consistency = 1.37 and rescue ratio = 2.32 for subsequent analyses (39, 40).

The FRiP score is defined as the fraction of all mapped reads that fall into peaks. This method is initially used as a measure of ChIP-seq quality and has been extend to ATAC-seq quality control. Most (74.81%) ENCODE ChIP-seq data sets have a FRiP score of 0.01 or more, and FRiP falling below 0.01 will be scrutinized (38). ENCODE defines that ATAC-seq FRiP score should be >0.3, though values >0.2 are acceptable. Ross et al. reported that ATAC-seq FRiP scores of livestock various tissues fall in 0.03-0.33 (37). Zhao et al. found that they ranged from 0.19 to 0.41 for pig different tissues across five breeds (41). The maximum of FRiP scores of our four samples was 0.13 and the minimum was 0.08. It has been demonstrated that FRiP score correlates positively and linearly with the number of called peaks (*r*^2^ = 0.67) (38). The lower sequencing depth and higher PCR duplication may make less peaks, which may partially lead to higher signal-noise ratio. Notably, FRiP scores may not be enforced as quality control metric for some exceptions, such as ENTEx tissues. Based on the above discussions, the qualities of our ATAC-seq libraries and sequencing data are acceptable but not ideal.

A total of 19,210 (C-6-2, adult), 20,361 (D-1-1, adult), 23,618 (T-6-2, embryo), and 24,550 (T-7-1, embryo) peaks were detected. These peaks produced 10,023 and 11,360 reproducible peaks for adult and embryo replicates, respectively. The two peak sets are reliable given the following facts: (1) PCA placed the four samples into two groups; (2) intra-group correlation coefficients (0.89–0.93) were substantially higher than that of different groups (0.31–0.47); (3) similar distributions of the two peak sets were observed. The two common peak sets were combined to form a union peak set which contained 8,850 DARs (7,422 embryo-specific peaks and 1,428 adult-specific peaks) and 10,342 stable peaks. A total of 2,515 promoter peaks were annotated from the 8,850 DARs. These promoters were involved in the cell cycle, muscle development, or lipid metabolism, as indicated by GO analysis. A total of 4,319 putative enhancers (3,494 embryo-specific and 825 adult-specific) were revealed by integrating the remaining 6,335 DARs with functional elements annotated by Nguyen et al. (8) and the ENCODE enhancer-like regions database. These regions were associated with muscle development and lipid metabolism, as revealed by GREAT and motif enrichment analyses. Detailedly, the embryo-specific putative enhancers were involved in abnormality of the liver, global developmental delay, and skeletal muscle atrophy (top 20 items of GREAT), while the adult-specific putative enhancers were annotated in muscle weakness, myopathy, and abnormality of muscle morphology (top 5 items of GREAT). SPs and KLFs are a family of transcription factors that contain three carboxyl-terminal (C-terminal) C2H2-type zinc finger structural motifs that bind to the GC-rich regions in DNA and regulate physiological processes such as growth, development, differentiation, proliferation, and embryogenesis (42). Notably, KLF family has been expanded to also include the SP transcription factors, forming the SP/KLF family (43). The roles of SP/KLF family in skeletal muscle growth and development have been well-documented, such as mediating extracellular signal-regulated kinase (ERK) 5 and FGFR1 and targeting TGF-β and MEF2A (31, 35, 36). Despite of the presences of SP/KLF family motifs in both adult and embryo groups, SP1, SP2, and SP3 were at the top of embryo AME list, while that were MEF2 family (2A, 2B, 2C, and 2D) in adult group. With the help of RGD database (http://animal.nwsuaf.edu.cn/code/index.php/RGD/), we found that the expressions of MEF2A and MEF2D in adult cattle muscle were significantly higher than that in embryo cattle muscle, but no differences were observed for MEF2B and MEF2C. It has been demonstrated that conditional deletion of the individual MEF2A, 2C or 2D genes in mouse satellite cells *in vivo* has no effect, but the combined conditional deletion of all three genes results in a block to regeneration (44, 45). These results provide unequivocal evidence that MEF2 is essential for muscle regeneration. Compared with MEF2A, 2C and 2D, MEF2B has the most divergent structure and the least evidence for involvement in muscle development, but significant association has been reported between MEF2B single nucleotide polymorphism (SNP) and sheep body weight (46).

We selected 15 enhancers for validation by focusing on well-known genes. Most of them (12/15) showed luciferase activity in cattle myoblasts, and their human homologous regions (8/12) have long-range interactions with gene promoters, such as peak1053 with *LCORL* and peak4218 with *FRMD8*. The *NCAPG* (non-SMC condensin I complex subunit G) *-LCORL* region is a major and pleiotropic locus for different economic traits, such as body weight, carcass weight, and stature (47). LCORL is a transcription factor and associated with skeletal frame size and the height of humans and horses (48, 49). FRMD8 could negatively regulate the Wnt pathway (50). These results suggest that complex transcriptional regulation at the 3D genome level was present at these loci. Therefore, chromosome conformation capture should be performed to confirm these interactions in the cattle muscle genome.

Mutations in enhancers have been found to be involved in complex traits or diseases of plants (4), livestock (51–53), and humans (5). These mutations are slightly deleterious and predisposed to escape natural selection, allowing them to reach an intermediate frequency (54). Therefore, enhancer mutations are promising candidates for beef cattle breeding. To identify GWAS hits associated with enhancers, we annotated the 4,319 enhancers with 3,635 GWAS hits regarding meat and carcass traits and production traits (trait classes of Animal QTLdb). We used 20 kb as the linkage disequilibrium (LD) cutoff. The extent of LD (present as *r*^2^) ranges from 0.2 to 0.5 for marker pairs separated by 20 kb but drops rapidly below 0.2 for markers with distances above 50 kb in various cattle breeds (55). Finally, 295 GWAS hits were mapped to 183 putative enhancers (140 embryo-specific and 43 adult-specific putative enhancers), such as rs109554838 to peak1053, rs110294629 to peak4218, and rs110066813 to peak1368. The GWAS hit rs109554838 located at 5′UTR of *NCAPG* has been reported to be associated with cattle body and average daily gain. The embryo-specific peak4218 was annotated to be involved in spasticity, hypertonia, abnormality of the liver by GREAT and rs110294629 mapped to it was associated with shear force and tenderness score. Peak4218 is in an intergenic region, and its human homologous region could interact with more than 18 gene promoters. This enhancer has been demonstrated to be involved in mouse embryo development (VISTA Enhancer ID: hs1759). Putative enhancer peak1368 is located at an intron of arrestin domain-containing 3 (*ARRDC3*) and near the *ARRDC3-AS1* (*ARRDC3* antisense transcript) promoter. This enhancer was downstream of rs110066813 by only 121 bp. The embryo-specific peak1368 was annotated to be involved in pigmentary retinopathy by GREAT and rs110066813 mapped to it was associated with average daily gain and length of productive life. Interestingly, the GeneHancer database indicates that peak1368 could contact the *ARRDC3* gene promoter (ENSG00000241059). ARRDC3 could interact directly with β-adrenergic receptors and, thus, regulate body mass and energy expenditure (56). Indeed, our comparative enhancer map is incomprehensive, given the spatiotemporal activity of enhancers during muscle development [(3), (57)].

## Conclusion

With ATAC-seq, we generated a comparative enhancer map for muscle genomes of adult and embryo cattle. This map might provide further insights into the genetic architecture of cattle muscle and, therefore, benefit beef cattle breeding.

## Data Availability Statement

The datasets presented in this study can be found in online repositories. The names of the repository/repositories and accession number(s) can be found in the article/Supplementary Material.

## Ethics Statement

The animal study was reviewed and approved by Institutional Animal Care and Use Committee of Northwest A&F University (NWAFAC1023).

## Author Contributions

XC and HC designed this study. XC conducted vector construction and cell culture. JC performed luciferase reporter assay. YH, XL, and CL gave instruction for English writing. All authors contributed to the article and approved the submitted version.

## Funding

This work was supported by the National Natural Science Foundation of China [Grant No. 31972558], the Agricultural Improved Seed Project of Shandong Province [Grant No. 2020LZGC014], and Program of National Beef Cattle and Yak Industrial Technology System [Grant No. CARS-37].

## Conflict of Interest

The authors declare that the research was conducted in the absence of any commercial or financial relationships that could be construed as a potential conflict of interest.

## Publisher's Note

All claims expressed in this article are solely those of the authors and do not necessarily represent those of their affiliated organizations, or those of the publisher, the editors and the reviewers. Any product that may be evaluated in this article, or claim that may be made by its manufacturer, is not guaranteed or endorsed by the publisher.

## Abbreviations

ARRDC3: arrestin domain-containing 3 protein
ATAC-seq: transposase-accessible chromatin using sequencing
DAR: differentially accessible regions
DCAF16: DDB1 and CUL4 associated factor 16
DNase-seq: DNase I hypersensitive site sequencing
EGR1: early growth response 1
ENCODE: Encyclopedia of DNA Elements
ERGIC1: endoplasmic reticulum-golgi intermediate compartment 1
FAANG: Functional Annotation of Animal Genomes
FADS1: fatty acid desaturase 1
FAIRE: formaldehyde-assisted isolation of regulatory elements
FAO: food and agriculture organization of the united nations
FASN: fatty acid synthase
FGFR4: fibroblast growth factor receptor 4
FRiP: fraction of reads in peaks
FRMD8: FERM domain containing 8
GO: gene ontology
GWAS: genome-wide association study
Hi-C: high-through chromosome conformation capture
HMGA2: high mobility group AT-hook 2
IGF2BP2: insulin like growth factor 2 mRNA binding protein 2
KLF15: kruppel like factor 15
IDR: irreproducible discovery rate
LCORL: ligand dependent nuclear receptor corepressor like protein
MEF2: myocyte enhancer factor 2
MNase-seq: micrococcal nuclease sequencing
NCAPG: non-SMC condensin I complex subunit G
PCA: principal component analysis
PLAG1: pleiomorphic adenoma gene 1
RRM: RNA-recognition motif
SDC3: syndecan 3
SH3PXD2A: SH3 and PX domains 2A
SP: specificity protein
TGFβ1: transforming growth factor β 1
TSS: transcription start site
UTR: untranslated region.
