# Supplementary material for: Comparative Enhancer Map of Cattle Muscle Genome Annotated by ATAC-Seq
Source: Front Vet Sci. 2021 Dec 15;8:782409. doi: 10.3389/fvets.2021.782409 (PMC8715921; doi:10.3389/fvets.2021.782409)
Supplement: Supplementary file 1 [file Data_Sheet_1.docx]

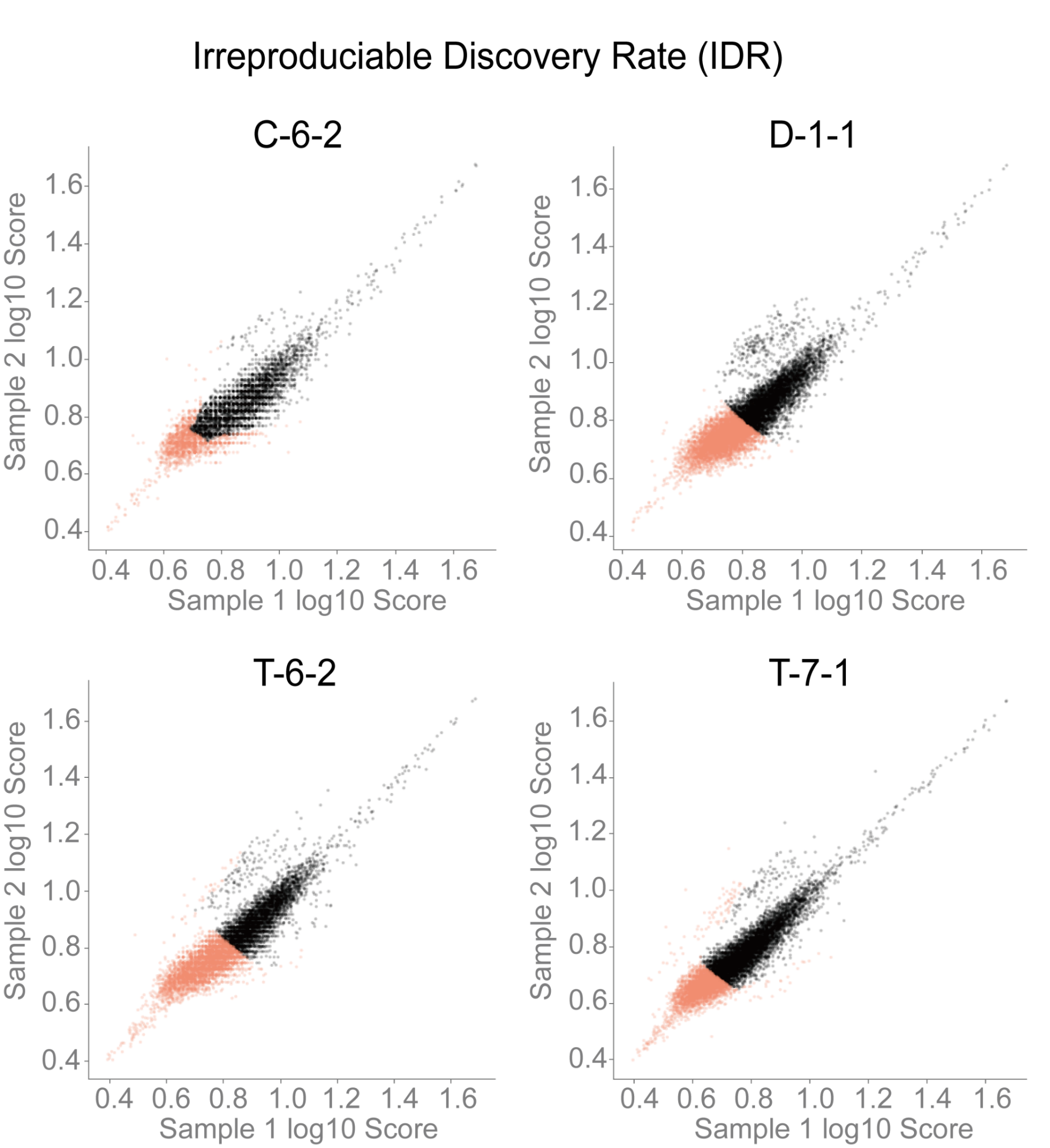


**Fig. S1** The visualization of peak reliability of each true replicate by producing pseudoreplicate peak set. x-Sample 1 log10 peak scores versus y-Sample 2 log10 peak scores. Peaks that did not pass the IDR threshold (0.05) were colored red. Sample 1 was the true replicate, and Sample 2 was pseudoreplicate (https://github.com/nboley/idr). Adult: C-6-2, D-1-1. Embryo: T-6-2, T-7-1.


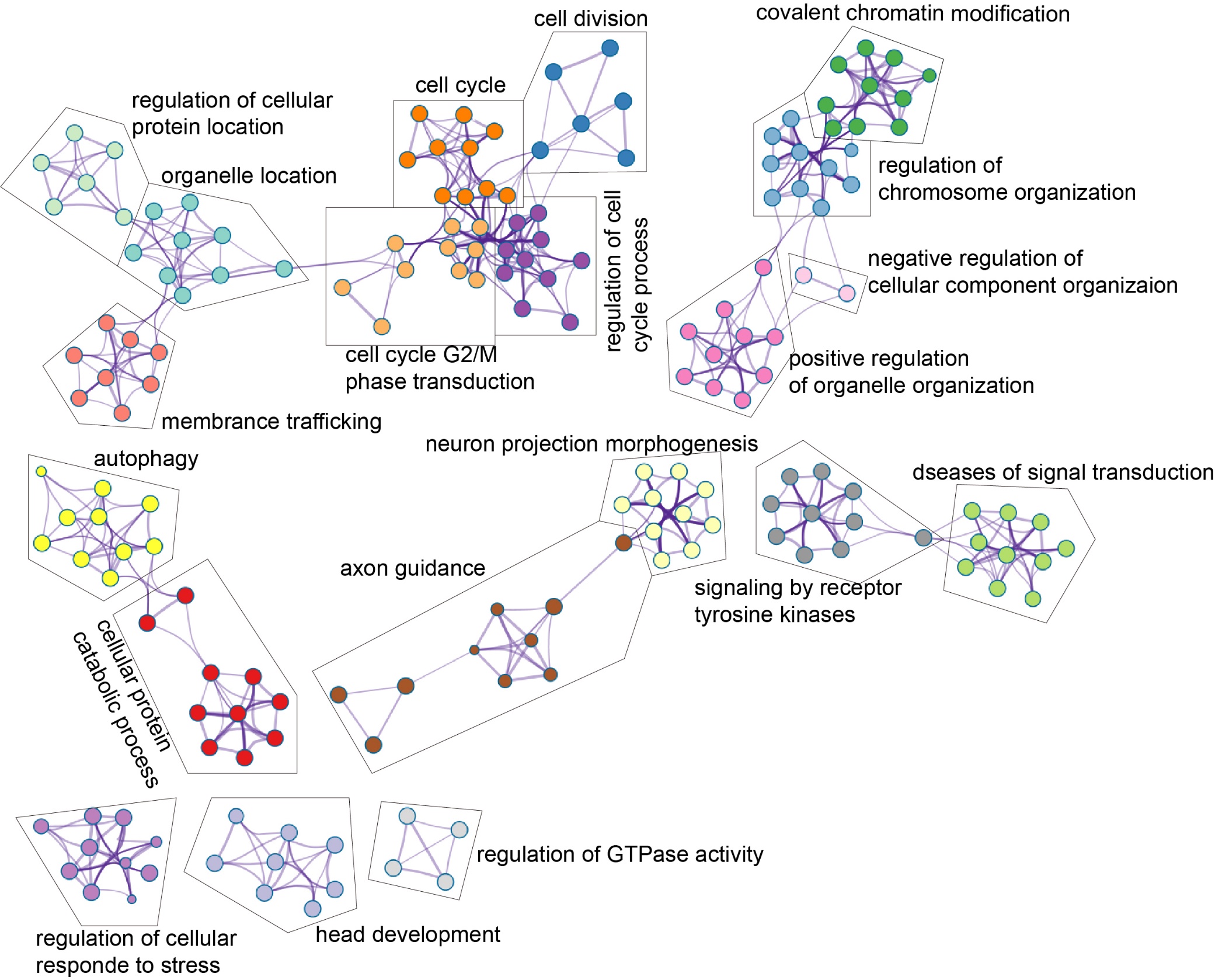


**Fig. S2** Top 20 GO items by Metascape of 2,515 genes with identified promoter peaks.


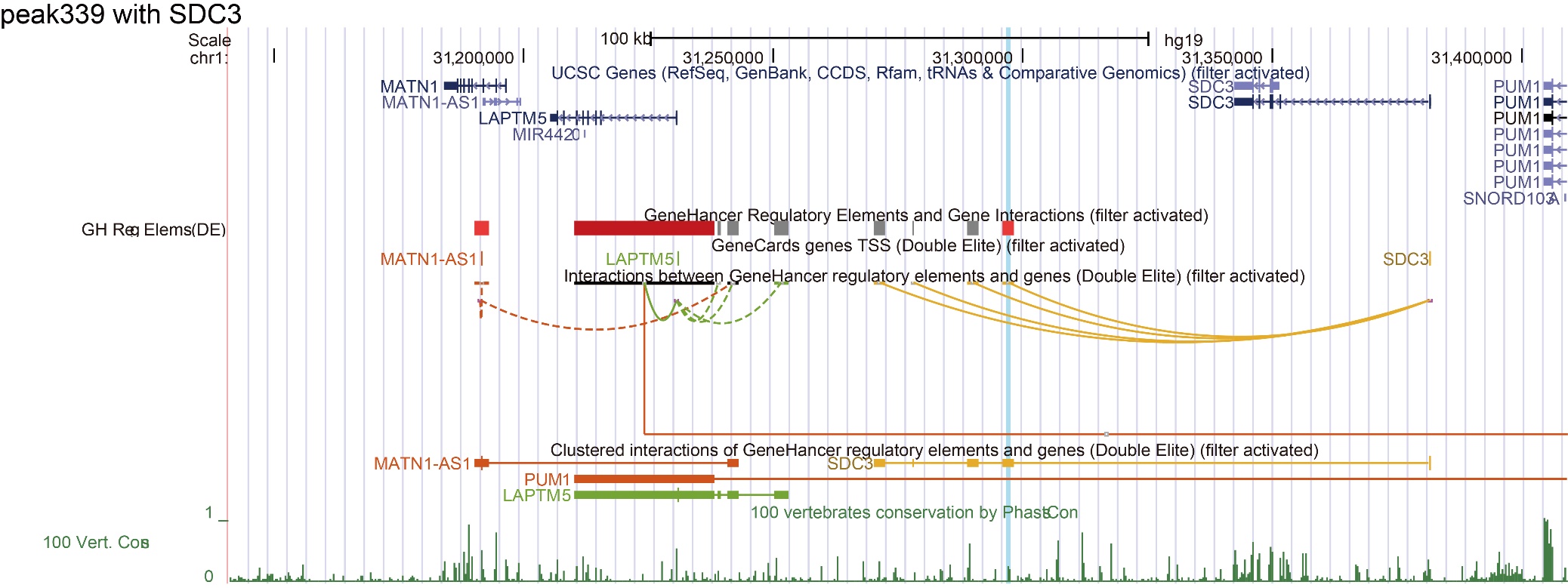


**Fig. S3-A** Peak339 interacts with *SDC3*.


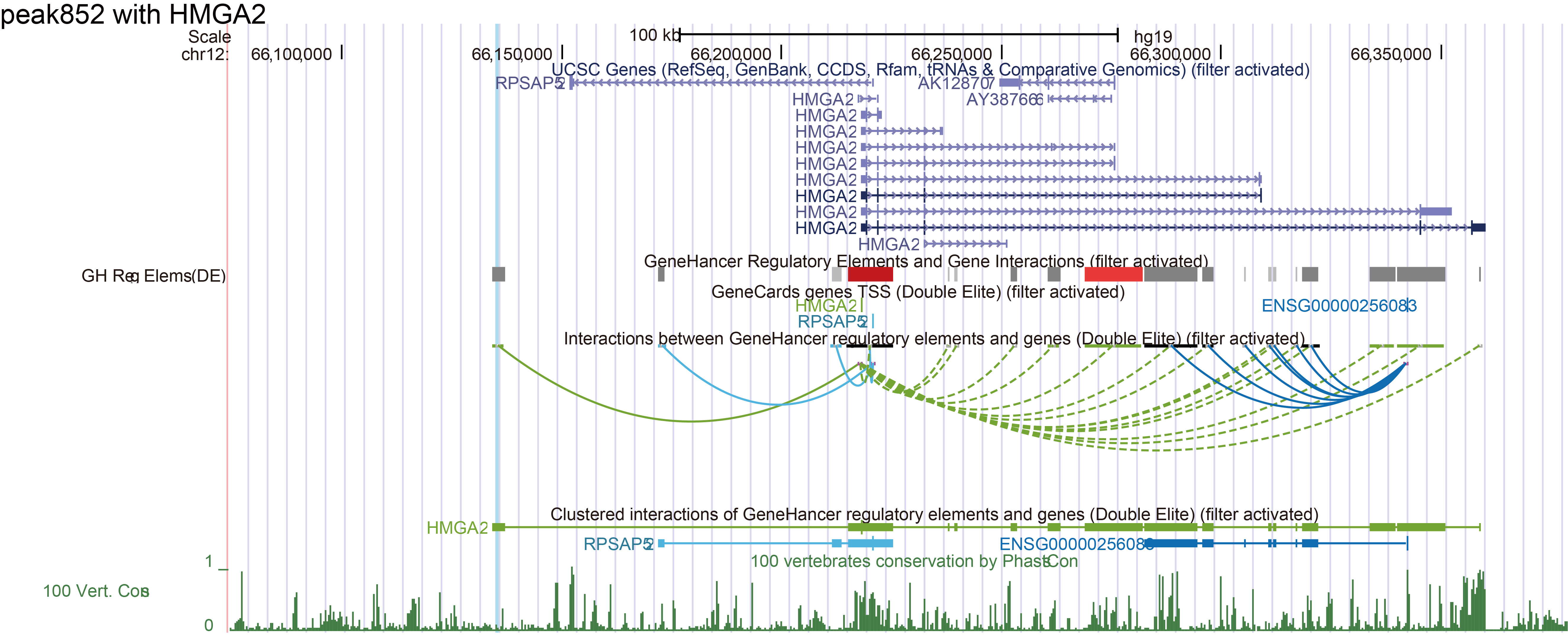


**Fig. S3-B** Peak 852 interacts with *HMGA2*.


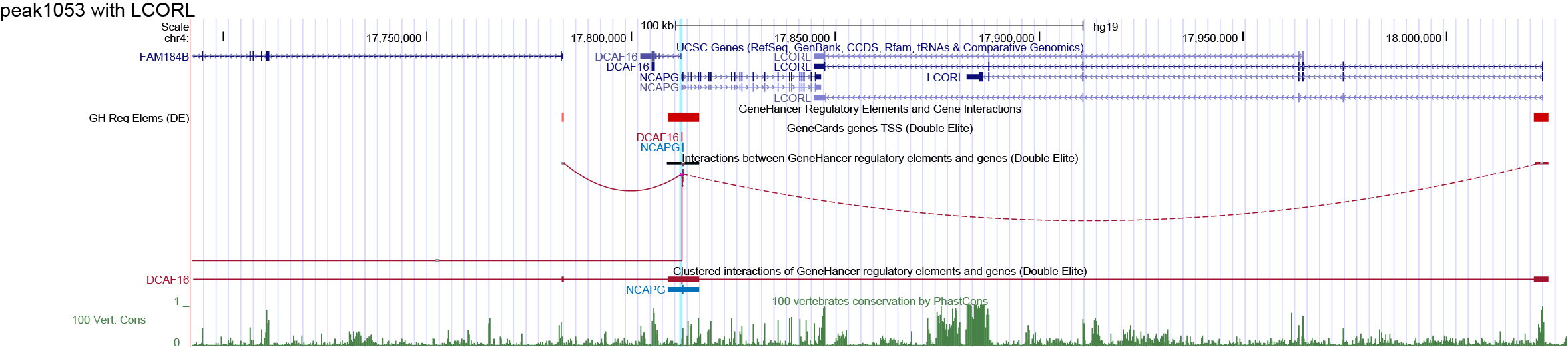


**Fig. S3-C** Peak1053 interacts with *LCORL*.


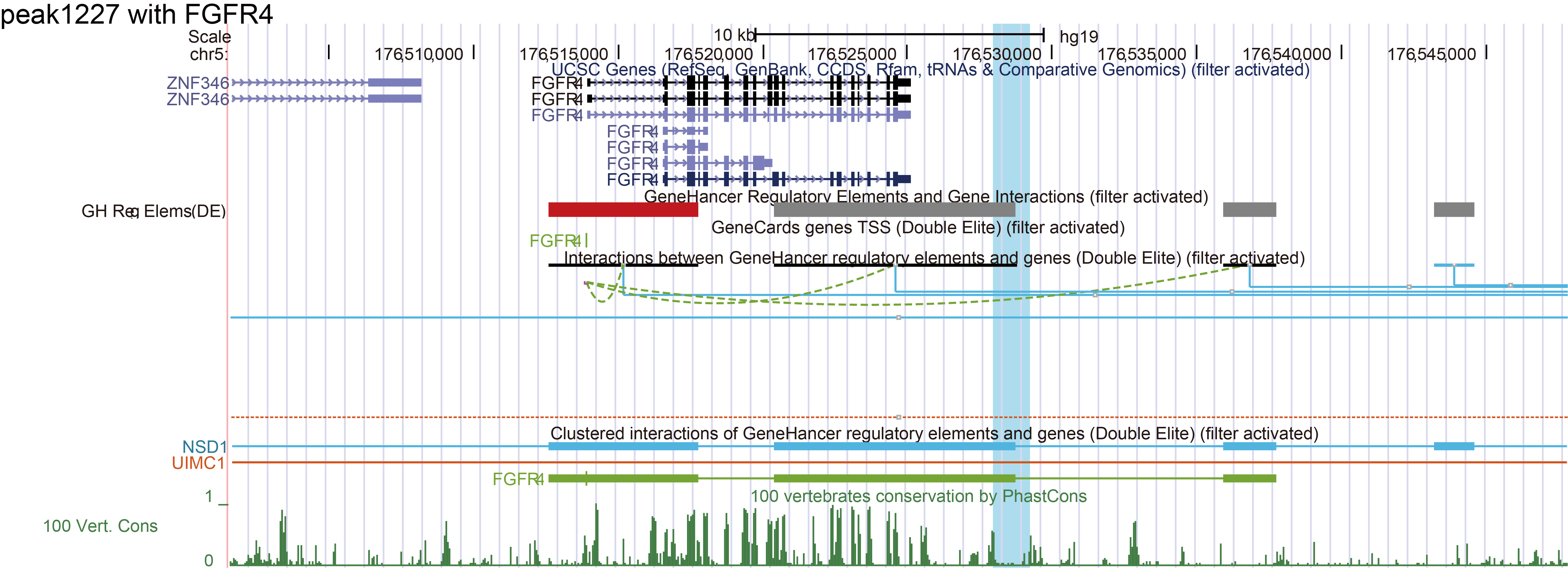


**Fig. S3-D** Peak1227 interacts with *FGFR4*.


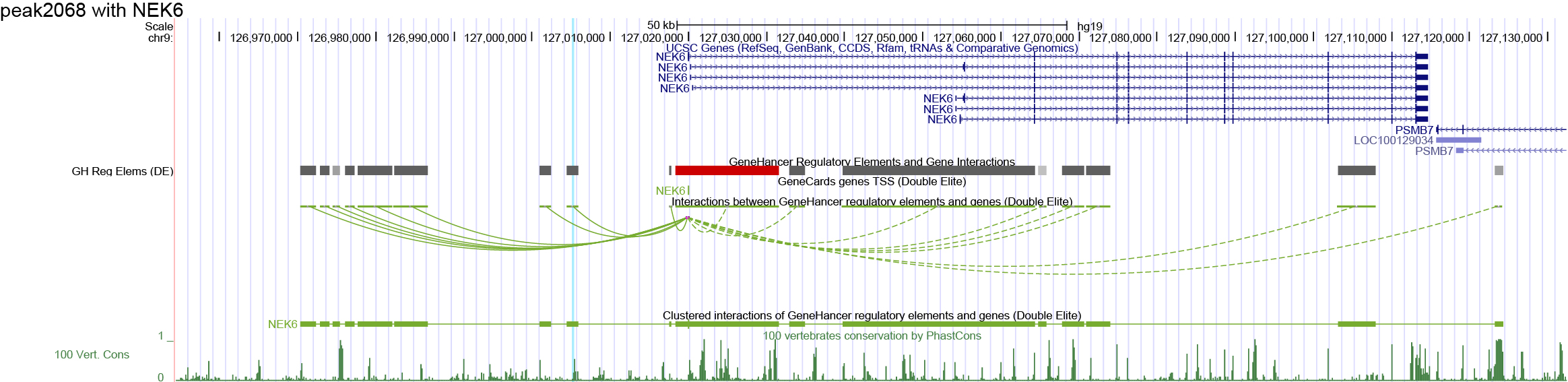


**Fig. S3-E** Peak2068 interacts with *NEK6*.


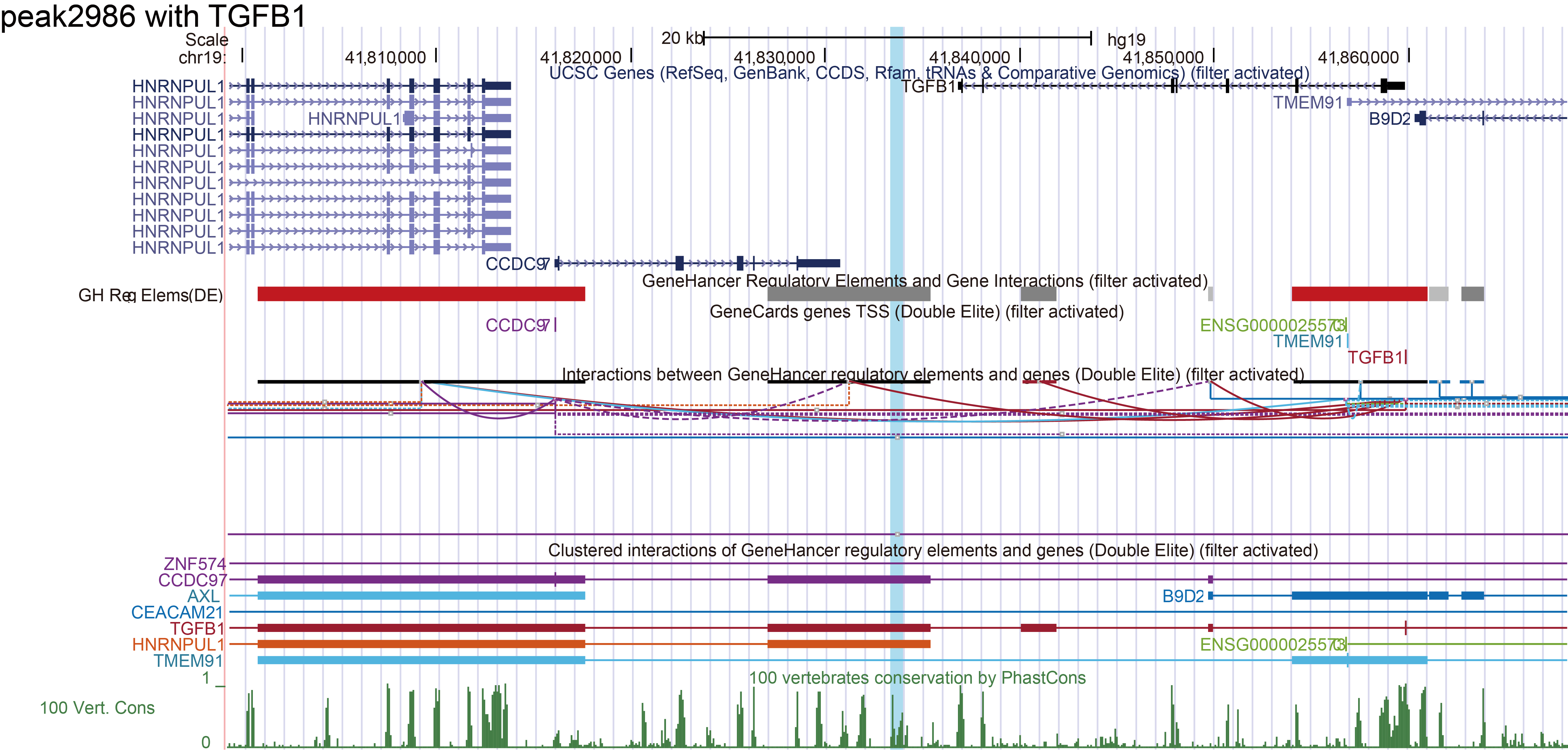


**Fig. S3-F** Peak2986 interacts with *TGFβ1*.


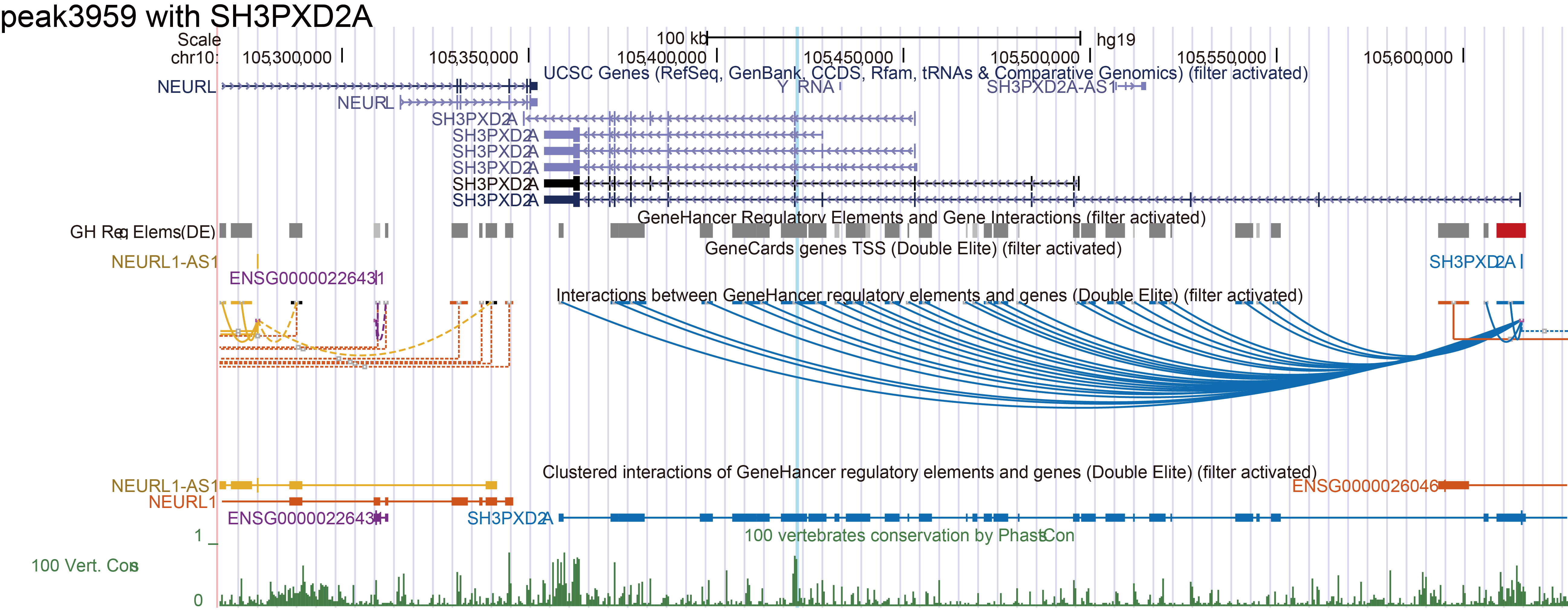


**Fig. S3-G** Peak3959 interacts with *SH3PXD2A*.
